# Ultra High-Performance Supercritical Fluid Chromatography for the Quantitation of Diterpene Resin Acids in Norway Spruce Samples

**DOI:** 10.3389/fphar.2022.906411

**Published:** 2022-06-13

**Authors:** Thomas Goels, Elisabeth Eichenauer, Julia Langeder, Georg F. Aichner, Gregor Mauser, Luisa Amtmann, Ulrike Grienke, Sabine Glasl

**Affiliations:** ^1^ Division of Pharmacognosy, Department of Pharmaceutical Sciences, Faculty of Life Sciences, University of Vienna, Vienna, Austria; ^2^ Vienna Doctoral School of Pharmaceutical, Nutritional and Sport Sciences, University of Vienna, Vienna, Austria

**Keywords:** *Picea abies*, Pinaceae, diterpene resin acids, quantitation, ultra high-performance supercritical fluid chromatography

## Abstract

*Picea abies* (L.) H. Karst. (Pinaceae) is native to Northern, Central and Eastern Europe. The fast-growing tree reaches up to 50 m in height, has modest nutritional requirements and depends on sufficient water supply. The conifer, commonly called Norway spruce, produces exudates which are traditionally used to treat skin wounds in Northern European countries. Major bioactive constituents of the conifer oleoresin are diterpene resin acids (DRAs) of the abietane and the pimarane type. To assure consistent pharmaceutical quality of Norway spruce balm and commercial products thereof, an analytical method for the quantitation of DRAs is the prerequisite. However, high structural similarity among DRAs and their poor UV absorption makes chromatographic separation and detection challenging: Conventional liquid chromatography systems often fail to achieve sufficient separation, moreover, they are not sustainable. Gas chromatography on the other hand requires time-consuming derivatization prior to unacceptably long analyses (>60 min). These drawbacks prompted the development of the first validated supercritical fluid-based protocol for the separation and quantitation of eight DRAs, i.e., pimaric acid (**1**), sandaracopimaric acid (**2**), palustric acid (**3**), isopimaric acid (**4**), levopimaric acid (**5**), abietic acid (**6**), dehydroabietic acid (**7**), and neoabietic acid (**8**). By using an ultra high-performance supercritical fluid chromatography (UHPSFC) device hyphenated to a quadrupole mass detector, the DRAs were separated in less than 20 min on a Torus 2-Picolylamin (2-PIC) column (3.0 mm × 100 mm; 1.7 µm particle size) applying supercritical CO_2_ and ethanol as mobile phase. Regarding selectivity, accuracy (recovery rates: 87–108%), intermediate precision (between 6.6 and 11.1%), and linearity (R^2^ ≥ 0.99; linear between 0.75 μg/ml and 2.5 mg/ml), results were obtained in line with ICH guidelines. The lowest limit of detection (LOD) was at 0.75 μg/ml (**7**) and the lowest limit of quantitation (LOQ) at 2 μg/ml (**8**). As application examples, 22 Norway spruce balm samples and five commercial products were analyzed. The here presented protocol not only simplifies and shortens the analytical workflow, but also reduces the amount of organic solvent waste by about two thirds compared to conventional liquid chromatographic set-ups. These advantages qualify this fast and efficient method as an ideal tool for an environmentally friendly quality control of traditionally used wound-healing Norway spruce balm products.

## 1 Introduction

**FIGURE 1:**
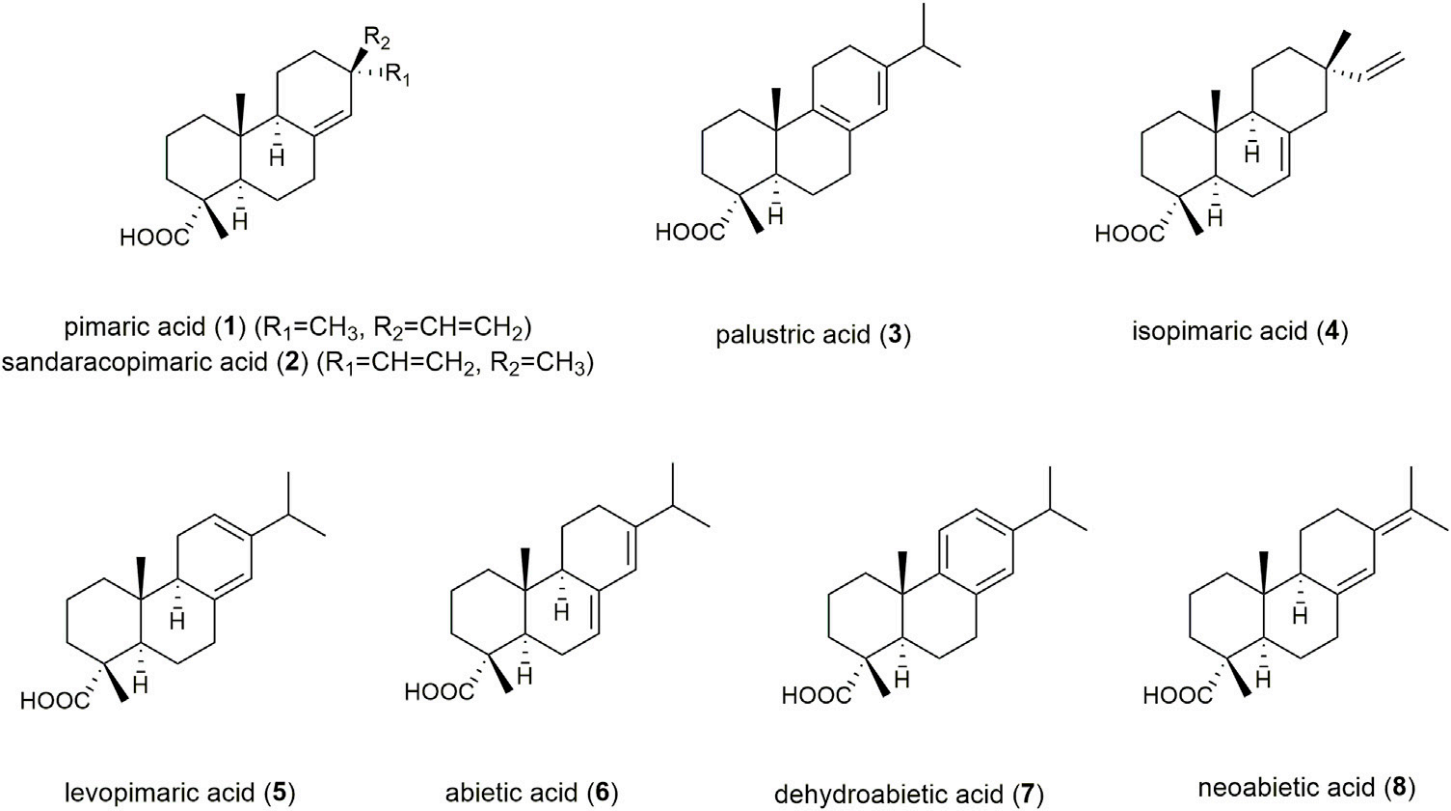
Structures of quantitated highly similar DRA congeners.

Coniferous trees produce highly viscous and adhesive exudates, in response to mechanical injuries, which provide protection against microbial infection and water loss (Holmbom et al., 2008; Kopaczyk et al., 2020). These exudates are complex mixtures consisting mainly of diterpene resin acids (DRAs), essential oil, lignans, and phenol carbonic acids (Keeling and Bohlmann, 2006; Holmbom et al., 2008; Metsämuuronen and Sirén, 2019; Kopaczyk et al., 2020). Fresh excretions are rich in essential oil, exhibiting paste-like and ductile characteristics with an aromatic odour. These fresh exudates are defined pharmaceutically as “balms” synonymously named “oleoresins” or “soft resins”, whereas “resins” result after evaporation of the essential oil, yielding non-volatile, brittle, and amorphous products soluble in nonpolar solvents (Burger, 1998). Coniferous tree balms have a long tradition in the treatment of infected and chronic wounds. The Ancient Greeks and Romans used coniferous excretions for wound dressings and hemostasis (Schmitz, 1998; Schnabl, 2001), and they have been continually used since the Middle Ages for their reputed curative effects. In Nordic countries, where the trees are in wide occurrence, ointments prepared from Norway spruce exudates have been among the most popular folk remedies for centuries, handed down from lumberjacks and others, who applied spruce excretions directly onto wounds (Pohl-Sennhauser, 1996). The application areas described, include infected wounds, sores, pressure ulcers, punctured abscesses, suppurating burns, onychomycosis and paronychia (Jokinen and Sipponen, 2016). Norway spruce balm and a lard-based ointment of the balm are monographed in the Austrian Pharmacopoeia (Österreichische Arzneibuchkommission, 2021). However, this monograph lacks precise parameters for quality control such as R_f_-values for marker compounds in TLC-fingerprints, or requirements of minimum contents for any class of compounds. DRAs play a major role in the wound healing activity of these natural products (Goels et al., 2020). For abietic acid (**6**), an angiogenic and migratory activity in human umbilical vein vascular endothelial cells (HUVECs) is proposed. The induced angiogenesis is associated with the upregulation of ERK and p38 expression, resulting in elevated cell migration and tube formation (Park et al., 2017). Dehydroabietic acid (**7**) was shown to reverse the tumour necrosis factor-α (TNF-α)/forkhead box O1 (FOXO1) and transforming growth factor-β1 (TGF-β1)/Smads mediated decrease in diabetic wound healing in human adult dermal fibroblasts (Wang et al., 2014). DRAs are therefore highly suitable as marker compounds for quality assessment of raw tree exudates (Goels et al., 2022). Efficient GC-MS based quantitation methods have already been described in the literature (Latorre et al., 2003; Holmbom et al., 2008; Alicandri et al., 2021). However, in addition to long analysis times (>60 min), this chromatographic technique requires sample derivatization prior to analysis in order to achieve volatility of the analytes. Although several authors focused on using HPLC with either UV- or MS-detection, their published chromatographic methods did not achieve sufficient separation of DRAs (Latorre et al., 2003; Kersten et al., 2006; Nilsson et al., 2008). Hence, the aim of this study was to overcome these drawbacks by developing a protocol using ultra-high performance supercritical fluid chromatography (UHPSFC) coupled with electrospray ionization mass spectrometry (ESI-MS) for the separation and detection of eight representative DRAs of the abietane and the pimarane type (Figure 1), pimaric acid (**1**), sandaracopimaric acid (**2**), palustric acid (**3**), isopimaric acid (**4**), levopimaric acid (**5**), abietic acid (**6**), dehydroabietic acid (**7**), and neoabietic acid (**8**). The eight specified diterpene acids possess high structural similarity, and thus exhibit highly similar chromatographic behaviour. The DRAs **1**–**6** and **8** have a molecular weight of 302 g/mol and differ solely in the position of one double bond and the side chain in position 13. DRA **7** has an additional double bond, resulting in an aromatic ring and a molecular weight of 300 g/mol.

The determining factors for the selection of an analytical setup were the properties of the targeted analytes (e.g., volatility, polarity etc.) and the availability of a suitable instrument. The high complexity and varying composition of samples from natural sources (e.g., due to different growth conditions) also presented a challenge. In this context, GC and HPLC are favoured techniques resulting from their broad availability and well-known chromatographic features. However, since the first application of a supercritical fluid in a chromatographic setting in the 1960s, supercritical-fluid chromatography (SFC) has evolved into a valuable technique for the separation and analysis of natural products. As a result of technical advances and the increased availability of SFC instruments, the current amount of analysable compound classes has broadened and is no longer limited to non-polar constituents. The combination of comparatively rapid and highly efficient separation with simple sample preparation and mild separation conditions, are significant advantages of this technology. The application of supercritical CO_2_ enables the analysis of thermolabile and hydrolysable constituents. A reduced environmental impact due to shortened run times and the omission of organic solvents as mobile phase, is an additional benefit. Today, SFC and the advanced ultra-high performance SFC (UHPSFC) constitute a versatile technology providing high separation efficiency, access to a broad range of compound classes, with various stationary phase chemistries available and high compatibility with numerous detectors (Schretter et al., 2020; Ganzera and Zwerger, 2021; Langeder and Grienke, 2021). For these reasons, this first UHPSFC method for the quantitative analysis of DRAs is preferable to the previously suggested techniques. Direct application to the UHPSFC system provides a big advantage over laborious sample preparation methods such as silylation for GC, which costs time and represents an additional source of errors and non-reproducibility. Compared to HPLC, UHPSFC is superior in separation efficiency and organic solvent waste production.

The UHPSFC method developed was validated according to the ICH guidelines and proved to be suitable for the quantitation of DRAs in raw plant material (balms) and commercial products prepared from spruce balms.

## 2 Materials and Methods

### 2.1 Chemicals and Standard Compounds

Dehydroabietic acid (**7**) was purchased from TCI chemicals with a declared purity less than 70%. Prior to validation it was purified *via* flash chromatography using a “Puriflash Column 15 C_18_ HP 6G - 6.0 g” as stationary phase and a linear gradient of methanol and water starting at 40% methanol with a rate of 2% per min. Dehydroabietic acid eluted at 33.1 min, its purity was determined *via* GC-FID to be 99.3%. All other resin acids were purchased from various suppliers and additionally checked for their purity by GC-FID: Neoabietic acid (**8**) (batch number: C1518, Santa Cruz Biotechnology; declared purity: ≥99%, confirmed *via* GC-FID: 99.0%); pimaric acid (**1**) (batch number: QR13961, MP Biomedicals; declared purity: 84%, confirmed *via* GC-FID: 84.6%); isopimaric acid (**4**) (batch number: H0718, Santa Cruz Biotechnology; declared purity: ≥99%, confirmed *via* GC-FID: 98.3%); palustric acid (**3**) (batch number: 3-TAH-178-1, TRC Canada; declared purity: 96%); levopimaric acid (**5**) (batch number: G2618, Santa Cruz Biotechnology; declared purity: 99.87%, confirmed *via* GC-FID: 46.9%); abietic acid (**6**) (batch number: X25C012, Alfa Aesar; declared purity: >90%, confirmed *via* GC-FID: 74.1%). For the identification of sandaracopimaric acid, sandarac resin was analyzed *via* UHPSFC-ESI-MS (Supplementary Figure S1) and GC-FID (Supplementary Figures S2, S3), since the pure substance was not commercially available. This resin was obtained from *Tetraclinis articulate* (supplier: Die Kräuterdrogerie) and contains sandaracopimaric acid as one of the most abundant components (Sugimoto et al., 2006; Kononenko et al., 2017). Sandaracopimaric acid corresponds to compound **2** in the Norway spruce balm samples (NSB). Compounds **7** and **8** were used as external standards and quantitated directly, whereas compounds **1**–**6** were calculated as compound **8** due to their structural similarities.

### 2.2 Samples and Sample Preparation

All samples analyzed in this work are listed in Supplementary Table S1 indicating batch number, date of harvest, harvest location, and expiration date. As a reference, 1 g of each sample is stored at −20°C at the Division of Pharmacognosy, University of Vienna. Norway spruce balm samples were either collected from various locations at different timepoints in Austria or were obtained from an in-house repository (Supplementary Table S1). The trees from which the samples were harvested were identified by Thomas Goels, Sabine Glasl or the in-house botanist Johannes Saukel. NSB01-NSB08 constitute samples from one single tree taken on the same day, but at eight different lesion sites. For NSB09-NSB22 different individuals were harvested. The samples were stored at −20°C prior to further processing. Before analysis, each sample was dissolved in acetone and filtered to remove cellular debris and other foreign material (e.g., insects, tree needles, pieces of bark). After removing the acetone under reduced pressure, the samples were re-dissolved in a mixture of *n*-hexane and isopropanol (50 + 50) at a concentration of 2 mg/ml. Two commercial products containing Norway spruce balm at concentrations of 10% (NSB-CP1, NSB-CP2 and NSB-CP3) and 20% (NSB-CP4 and NSB-CP5) were analyzed. The samples varied in batch numbers and storage conditions. (Supplementary Table S1). Both preparations were dissolved at a concentration of 10 mg/ml. For NSB-CP4 and NSB-CP5 a mixture of *n*-hexane and isopropanol (50 + 50) and for NSB-CP1, NSB-CP2 and NSB-CP3 pure hexane was used as solvent. All reference compounds were dissolved in a mixture of *n*-hexane and isopropanol (50 + 50) at varying concentrations. For each solution (balm, commercial product, reference), 1 µl was applied to the UHPSFC-MS system.

### 2.3 Analytical Method

The UHPSFC instrument Acquity UPC^2^ (ultra-performance convergence chromatography) from Waters was used, which consists of a sample manager (sample temperature: 8°C), convergence manager, binary solvent manager (flow: 1 ml/min), isocratic solvent manager (10 mM ammonium formate in MeOH-H_2_O (95 + 5); flow: 0.6 ml/min) and a column manager (column temperature: 40°C). A Waters Acquity QDa single quadrupole mass detector (mass range 150–700 Da, cone voltage positive scan 15 V, negative scan 30 V, capillary voltage positive 0.8 kV, negative 0.8 kV) was used. The mass chromatograms used for the integration of peak areas were deduced from the negative ionization mode. A Torus 2-Picolylamin (2-PIC) column (3.0 mm × 100.0 mm, 1.7 µm) served as stationary phase. The mobile phase consisted of supercritical CO_2_ with ethanol as co-solvent. The separation was achieved by applying a gradient of the co-solvent starting from 0 to 3% ethanol within 8 min (hold for 2 min) and then to 5.5% within 5 min. A final washing step (50% ethanol for 1 min and 0% ethanol for 1 min) marked the end of the gradient.

### 2.4 Method Validation and Quantitation

In a first step, the main DRAs in the samples were identified by co-chromatography with commercially available references. Solutions of pimaric acid (**1**), palustric acid (**3**), isopimaric acid (**4**), levopimaric acid (**5**), abietic acid (**6**), dehydroabietic acid (**7**), and neoabietic acid (**8**) were prepared in a mixture of *n*-hexane and isopropanol (50 + 50) at a concentration of 0.75 mg/ml each. Sandarac resin was prepared the same way to identify sandaracopimaric acid (**2**). The method was validated in accordance with the ICH Q2 (R1) guideline (“Validation of analytical procedures”) (International Council for Harmonisation of Technical Requirements for Pharmaceuticals for Human Use (ICH), 1995) using dehydroabietic acid (**7**) and neoabietic acid (**8**) as external standards and sample NSB22 as herbal material. The choice of **7** was made due to its aromatic structure with a molecular weight of 300 g/mol, **8** represented the abietic-type acid with a molecular weight of 302 g/mol. The linearity was determined by preparing solutions of the standards in *n*-hexane and isopropanol (50 + 50) at nine different concentrations (7.5, 2.5, 0.75, 0.25, 0.075, 0.025, 0.0075, 0.0025, and 0.00075 mg/ml, Supplementary Tables S2, S3 and Supplementary Figures S4, S5). A sample of 1 µl was injected into the chromatographic system. The specificity was examined comparing the retention times and *m/z* values of the pure compounds in a concentration of 0.75 mg/ml to the respective substance in sample NSB22 (2 mg/ml, Supplementary Table S4). The accuracy of the method was determined by spiking sample NSB22 (2 mg/ml) with three different concentrations of **7** (0.183, 0.244 and 0.305 mg/ml, Supplementary Table S5) and **8** (0.0013, 0.0017, 0.0022 mg/ml, Supplementary Table S6). For the precision experiments three independent solutions of sample NSB22 at three different concentrations (0.625, 1.25, and 1.875 mg/ml) were prepared. Mean values, standard deviation and relative standard deviations of three independently prepared sample solutions were calculated for each of the eight diterpene acids to measure their reproducibility (Supplementary Table S7). The intermediate precision was assessed by injection of the highest concentration (1.875 mg/ml) in triplicates on three consecutive days (Supplementary Tables S8, S9). For LOD and LOQ the values of the noise were recorded as peak heights at ten different time points in an analysis of a solvent blank and calculated as a mean value (Supplementary Tables S10–S13).

The contents of all diterpene acids, except for dehydroabietic acid, in the samples were related to neoabietic acid and calculated according to following formula:

$$\%\;analyte=\frac{amount_{\mathrm{standard}}\;\times \;AUC_{analyte}\;\times \;100}{AUC_{\mathrm{standard}}\;\times \;weight_{sample}}$$

## 3 Results and Discussion

### 3.1 Sample Preparation

Since Norway spruce balm is a conifer exudate without cellular components, no conventional extraction method was necessary. The crude balm had only to be separated from foreign material (e.g., tree bark particles), which was accomplished by a dissolution and filtering step. Different solvents (heptane, hexane, ethyl acetate, methanol, ethanol, acetone, chloroform, dichloromethane) were tested, with acetone and dichloromethane found to be the most efficient solvents. Acetone was selected on the basis of low toxicity, ease of waste management and low adverse environmental impact.

### 3.2 Method Development

An enriched extract containing the DRAs **1**–**8** was prepared for the optimisation of the UHPSFC analysis. Method development was accomplished in three steps: 1) rapid column and co-solvent screening with a generic gradient, 2) defined gradient optimization with a specified stationary phase chemistry and co-solvent, and 3) detailed optimization regarding flow rate, column temperature, back pressure, and mass detector parameters including make-up solvents for ionization. As an initial step, eight columns with different stationary phase chemistries were tested for their separation capacity, by employing a generic gradient from 0 to 50% co-solvent (methanol). Out of the eight stationary phases tested (Waters Torus series: 1-Aminoanthracene (1-AA, 1.7 μm), 2-Picoylamine (2-PIC, 1.7 μm), Diethylamine (DEA, 1.7 μm), High-density Diol (DIOL, 1.7 μm); Waters Vidiris series: Bridged-Ethylene-Hybrid (BEH, 1.7 μm), Bridged-Ethylene-Hybrid 2-Ethylpyridine (BEH 2-EP, 1.7 μm), Charged-Surface-Hybrid Fluoro-Phenyl (CSH FP, 1.7 μm), Silica 2-Ethylpyridine (Silica 2-EP, 5 μm); dimension for each column 3.0 × 100 mm), the 2-Picoylamine column showed the highest potential. In a subsequent step, four co-solvents (methanol, ethanol, isopropyl alcohol, acetonitrile) were screened with CO_2_ on the 2-PIC column for the best suitability. Ethanol achieved a satisfactory separation and was used for further gradient optimization. Starting with a linear gradient from 0% to 50% ethanol, different variations were examined. The final gradient is depicted in Table 1. As a last step the effect of varying the flow rate, the temperature of the column, the backpressure and the use of different make-up solvents for ionization was analyzed. For an improved ionization for mass detection of the QDa detector, a make-up solvent is necessary. Of the three employed make-up solvents (95% MeOH + 5% H_2_O with 10 mM ammonium formate; 95% MeOH + 5% H_2_O with 10 mM ammonium acetate; 99% MeOH + 1% H_2_O with 0.1% formic acid) the mixture with ammonium formate showed the best results. The variation of the other named parameters had no advantageous effect on the peak shape or resolution and were therefore kept to the values listed in Table 1.

**TABLE 1:** Optimized UHPSFC method settings.

Parameter	Value		
Injection volume	1 µl		
Sample temperature	8°C		
Co-solvent A	CO_2_		
Co-solvent B	Ethanol		
Gradient	Time (min)	%A	%B
	0	100.0	0.0
	8	97.0	3.0
	10	97.0	3.0
	15	94.5	5.5
Stationary phase	Torus 2-Picolylamin (3.0 mm × 100.0 mm, 1.7 µm)		
Column temperature	40°C		
Flow rate	1.00 ml/min (BSM)		
	0.60 ml/min (ISM)		
Detection	QDa (neg. mode)		
Make-up solvent	10 mM ammonium formate in MeOH-H_2_O (95 + 5)		
Back-pressure	2000 psi		

**FIGURE 2:**
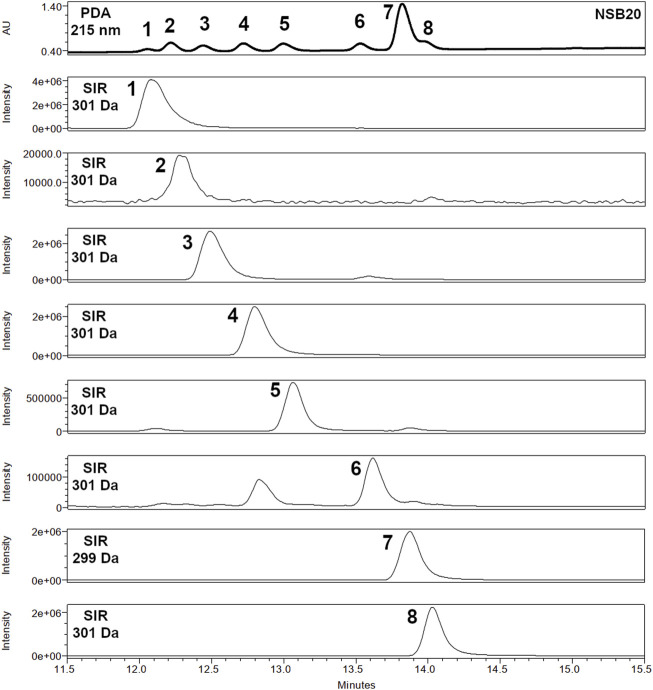
Detailed section (RT 11.5–15.5 min) of the PDA chromatogram of spruce balm sample NSB20 (at 215 nm) and UHPSFC-ESI-MS Extracted Ion Chromatograms (negative mode, 299 and 301 Da) of diterpene resin acids **1**–**8** with optimized parameters on a 2-PIC column (3.0 mm × 100.0 mm, 1.7 µm).

Figure 2 illustrates the analysis of the balm sample NSB20 and the available reference substances (**1**–**8**) with the final chromatographic method. A UHPSFC-MS and GC-FID chromatogram of sandarac resin containing mainly compound **2** in comparison to the balm sample NSB22 is available in the supporting material (Supplementary Figures S1, S2).

### 3.3 Method Validation

To ensure the suitability of the generated protocol for the quantitation of compounds **1**–**8** in Norway spruce samples using mass detection, a validation according to ICH guidelines (International Council for Harmonisation of Technical Requirements for Pharmaceuticals for Human Use (ICH), 1995) was performed. The raw data from the validation is listed in the supporting information under Section 3. Results shown in Table 2 including evaluation of linearity, limit of detection (LOD), limit of quantitation (LOQ), as well as precision and accuracy are in accordance with the ICH recommendations.

**TABLE 2:** Validation results.

	7	8
Regression equation	y = 1E+08x—63000	y = 1E+08x—70000
R^2^	0.9992	0.9966
Linearity	0.00075 and 2.5 mg/ml	0.00075 and 2.5 mg/ml
LOD	0.00075 mg/ml	0.00100 mg/ml
LOQ	0.0025 mg/ml	0.0020 mg/ml
Accuracy		
Low spike amount	97.0% (±0.1% RSD)	104.1% (±4.5% RSD)
Medium spike amount	93.1% (±0.3% RSD)	105.6% (±8.0% RSD)
High spike amount	86.6% (±1.7% RSD)	107.8% (±4.9% RSD)
Precision		
Repeatability	0.625 mg/ml: 0.7 RSD	0.625 mg/ml: 6.4 RSD
	1.250 mg/ml: 0.9 RSD	1.250 mg/ml: 8.2 RSD
	1.875 mg/ml: 1.8 RSD	1.875 mg/ml: 3.8 RSD
Intermediate Precision	1.875 mg/ml: 10.5 RSD	1.875 mg/ml: 11.1 RSD
Specificity	−5.3 and +6.1 s	

### 3.4 Application to Balm Samples and Commercial Products

From the mass chromatograms recorded in the negative mode the ion chromatograms at *m/z* = 299 Da and *m/z* = 301 Da were extracted and used for integration of peak areas. The identification of the compounds was performed by comparing retention times and mass spectra with the references. Compounds **7** and **8** were directly quantitated, whereas compounds **1**–**6** were calculated as neoabietic acid (compound **8**).

**FIGURE 3:**
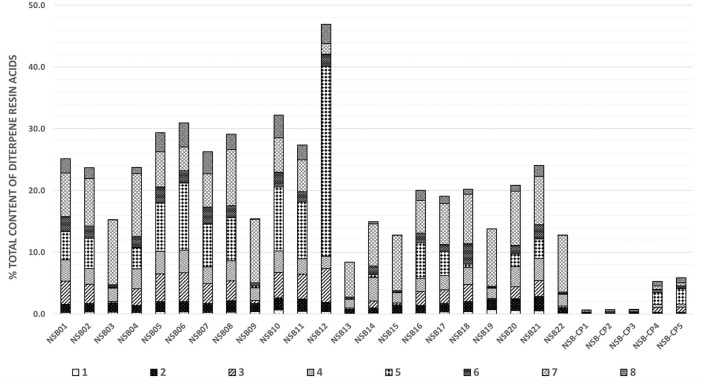
Total contents of DRAs (% ± SD) in Norway spruce balms (samples NSB01-22) and commercial products (NSB-CP1-5).

Figure 3 and Supplementary Table S14 show the quantitative composition of the investigated samples and confirm a substantial variation of the total DRA content in the raw balms ranging between 8 and 47%. Even samples harvested from one single individual on the same day from different injury sites (NSB01-08) show heterogeneity (15–31%). The main constituents in all samples are **5** and **7** (Supplementary Table S14), which is in accordance with the literature where samples of “*Picea abies* oleoresin” originating from Southern Finland had been analyzed by GC-FID (Holmbom et al., 2008). The two samples of the commercial product NSB-CP4 and NSB-CP5 are coherent. These samples were produced from Norway spruce balms originating from Lungau/Austria. Their composition approximatively matches the analyzed balms which had all been harvested in Austria. In contrast, the composition of the medical device NSB-CP1, NSB-CP2, and NSB-CP3 clearly differs in content and composition. The product is produced in Finland and claims to contain “resin” of Norway spruce. Use of the resin instead of the balm could be one explanation for these differences. Matrix effects could be another reason for unexplainable high or low values. The influence of co-eluting components, e.g., from the ointment base or other biological material on the ionization efficiency has not been investigated in this paper but needs to be evaluated in a next step.

## 4 Conclusion

UHPSFC was used to separate and quantitatively determine the main DRAs in *Picea abies*. To the best of our knowledge, this is the first time that UHPSFC has been used as a separation technique for spruce balms and commercial products containing such exudates. The method presented provides high selectivity and achieves separation of structurally similar DRAs where conventional RP-HPLC fails. Even though previously-employed GC analyses had shown better separation, they were disadvantageous with regard to sample preparation (e.g., derivatization) and duration of analysis (>60 min). With a run time of 17 min the optimised UHPSFC method is clearly superior to GC at acceptable separation performance. The minimized consumption of organic solvent and the resulting reduced ecological impact together with simple sample preparation, are significant advantages of this technology. Up until now there has been only an organoleptic examination and a TLC analysis required according to the monograph of the Austrian Pharmacopoeia (Österreichische Arzneibuchkommission, 2021). The method presented allows for the determination of DRAs in pharmaceutical matrices like Vaseline (NSB-CP1, NSB-CP2, and NSB-CP3) and lard (NSB-CP4 and NSB-CP5). Thus, it represents a basic tool for quality control providing the differentiation of balms from different species of the Pinaceae family (Goels et al., 2022). Moreover, it may serve as quantitation method, e.g., in *ex vivo* permeation experiments revealing information about the ability of DRAs to permeate skin into an acceptor medium. Such analyses require preceding spiking experiments to evaluate, whether matrix effects of compounds from biological material influence the detector response or whether such effects may be disregarded.

## Data Availability

The original contributions presented in the study are included in the article/Supplementary Material, further inquiries can be directed to the corresponding author.
