# Supplementary material for: Ultra High-Performance Supercritical Fluid Chromatography for the Quantitation of Diterpene Resin Acids in Norway Spruce Samples
Source: Front Pharmacol. 2022 Jun 13;13:906411. doi: 10.3389/fphar.2022.906411 (PMC9234136; doi:10.3389/fphar.2022.906411)
Supplement: Supplementary file 1 [file Presentation1.pdf]

Supplementary Material

1. Norway spruce balm samples

Batch #	Date of harvest	Harvest location	Commercial product	Batch #	Expiration date	Storage conditions
NSB01 - NSB08	09.07.2016	Promau Kitzhütte, Hollenstein, Lower Austria	NSB-CP1	552026	06/21	stored at room temperature
NSB09 - NSB12	03.07.2016	Gaflenz Heiligenstein, Upper Austria	NSB-CP2	552014	01/19	stored at room temperature
NSB13	2015	Lungau, Salzburg	NSB-CP3	552014	01/19	stored at 2 – 8°C
NSB14	15.05.2016	Zillingtaler Wald, Burgenland	NSB-CP4	160301	03/20	stored at room temperature
NSB15	15.05.2016		NSB-CP5	160301	03/20	stored at 2 – 8°C
NSB16	Spring 2016	Pinsdorf, Upper Austria				
NSB17	10.07.2015	unknown				
NSB18	24.08.2016	Lungau, Salzburg				
NSB19	Harvest 2010-2016	Austria				
NSB20	Harvest 2010-2016	Austria				
NSB21	Harvest 2010-2016	Austria				
NSB22	23.08.2017	Lungau, Salzburg				

Supplementary Table S1. Details for Norway Spruce Balm Samples NSB01-22 and Commercial Products Samples

2. Sample chromatogram

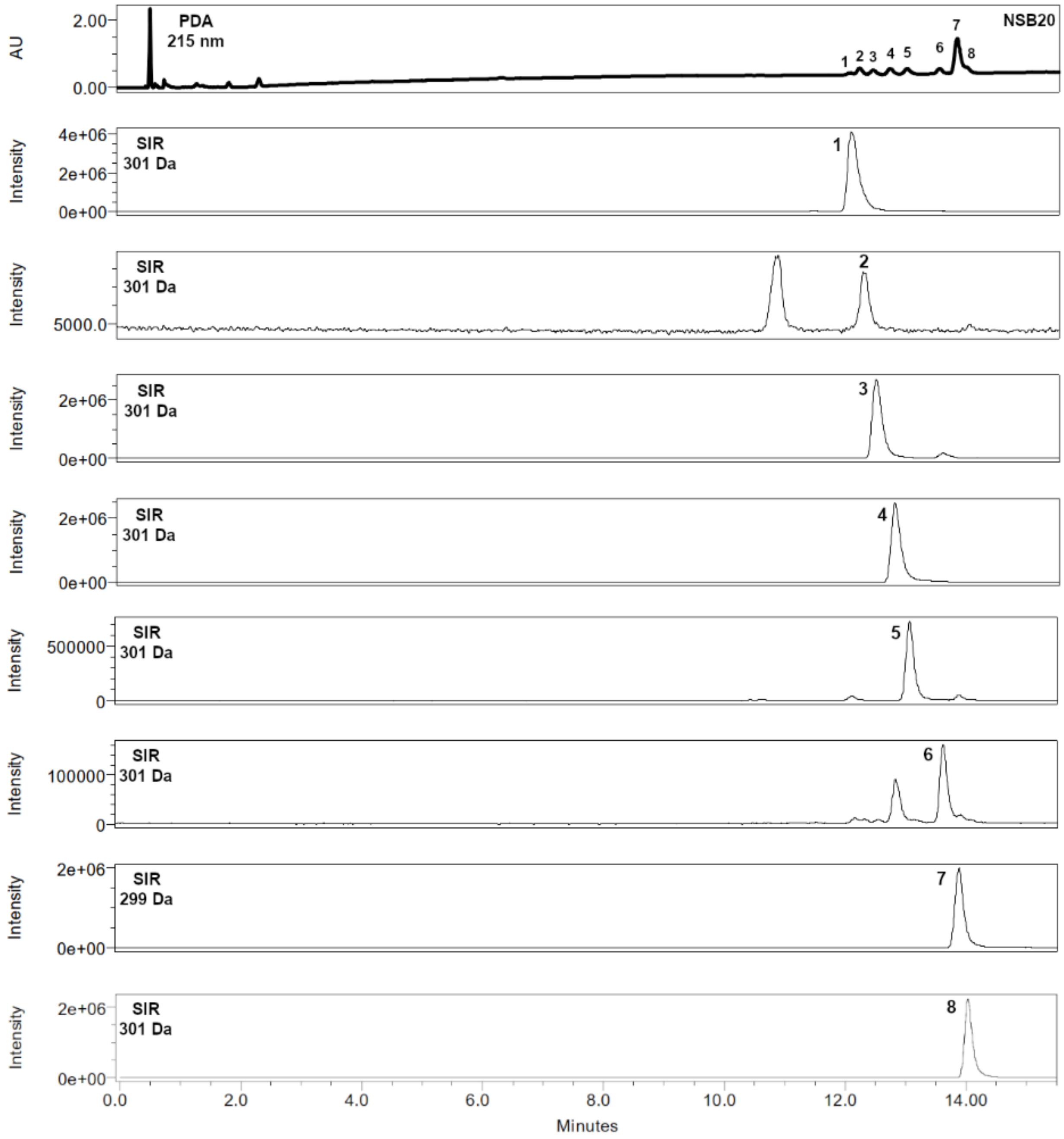

Supplementary Figure S1. PDA chromatogram of spruce balm sample NSB20 (at 215 nm) and UHPSFC-ESI-MS Extracted Ion Chromatograms (negative mode, 299 and 301 Da) of diterpene resin acids. Compound **1**: Pimaric acid, **2**: Sandaracopimaric acid (sample: sandarac resin), **3**: Palustric acid, **4**: Isopimaric acid, **5**: Levopimaric acid, **6**: Abietic acid, **8**: Neoabietic acid

3. Validation

3.1. Sandarac resin

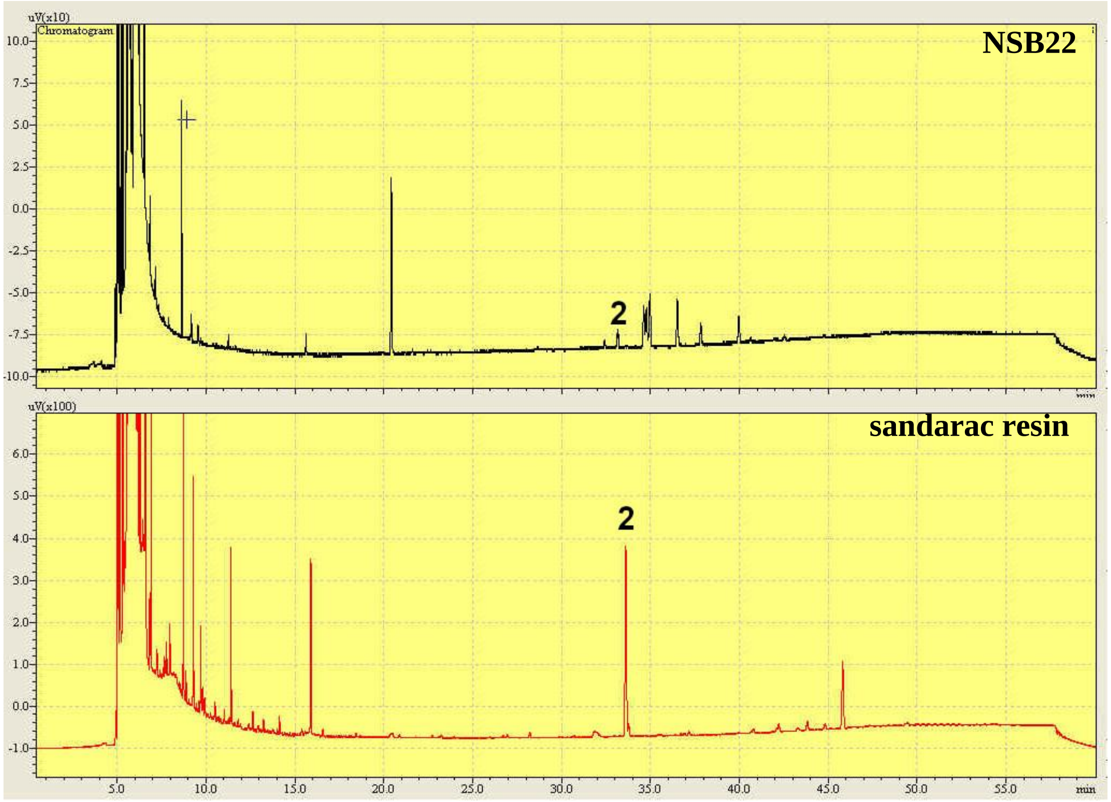

Supplementary Figure S2. GC-FID chromatogram of spruce balm sample NSB22 and sandarac resin. Compound **2**: sandaracopimaric acid. GC-System Shimadzu GC-2010: stationary phase ZB-5 Zebron, 60 m, 0.25 mm, 0.25 µm; mobile phase: He (5.0), flow: 50 ml/min; temperature program: 5 min 100°C, 100-200°C rate 15°C/min, 200-250°C rate 2°C/min; injection volume: 1 µl, split ratio 1:100; detection: synthetic air (5.0), H_2 (5.0)

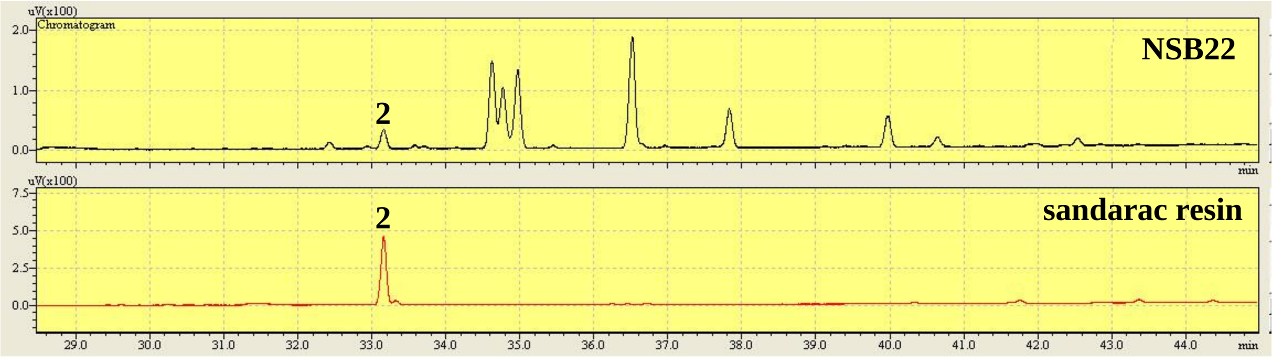

Supplementary Figure S3. Detailed section (RT 28.5-45 min) of GC-FID chromatogram of spruce balm sample NSB22 and sandarac resin. Compound **2**: sandaracopimaric acid. GC-System Shimadzu GC-2010: stationary phase ZB-5 Zebron, 60 m, 0.25 mm, 0.25 µm; mobile phase: He (5.0), flow: 50

ml/min; temperature program: 5 min 100°C, 100-200°C rate 15°C/min, 200-250°C rate 2°C/min; injection volume: 1 µl, split ratio 1:100; detection: synthetic air (5.0), H_2 (5.0)

3.2. Linearity

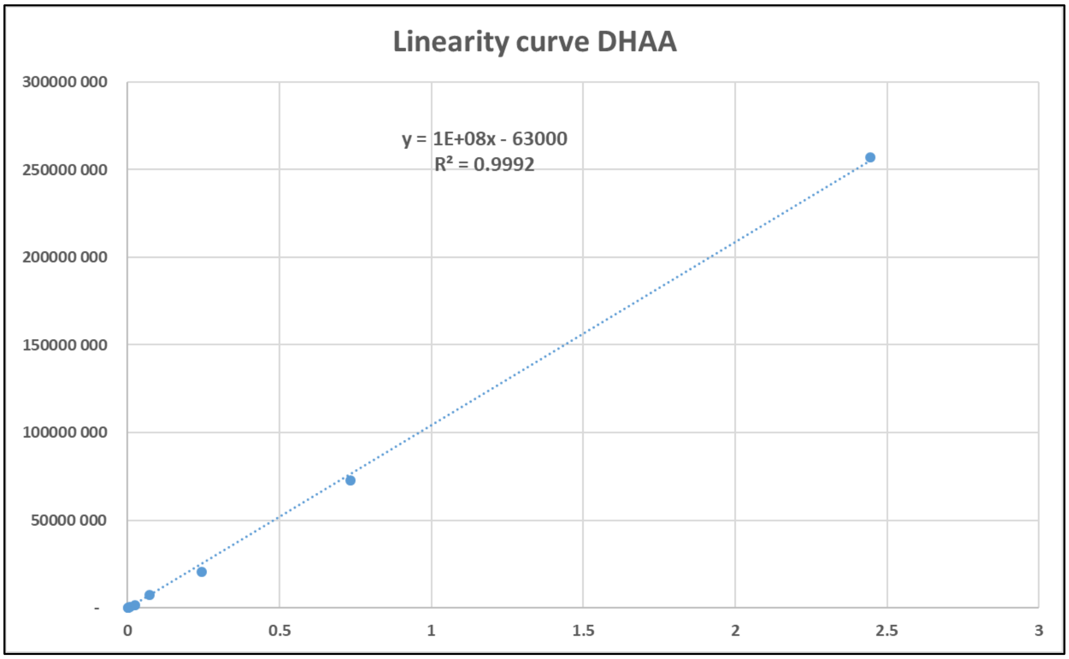

Supplementary Figure S4. Linearity curve of dehydroabietic acid

c [mg/ml]	Run	RT [min]	Area	Mean
7.335	1	13.725	475 897 268	466 371 025
	2	13.738	419 734 916	
	3	13.726	503 480 890	
2.445	1	13.766	255 190 971	257 088 347
	2	13.760	257 112 675	
	3	13.766	258 961 397	
0.7335	1	13.812	73 152 487	72 593 616
	2	13.823	72 625 126	
	3	13.808	72 003 234	
0.2445	1	13.870	19 252 645	20 360 947
	2	13.884	20 679 791	
	3	13.874	21 150 404	
0.07335	1	13.901	7 228 218	7 234 994
	2	13.914	7 313 775	
	3	13.904	7 162 989	

0.02445	1	13.927	1 602 098	1 641 123
	2	13.929	1 603 410	
	3	13.928	1 717 862	
0.007335	1	13.956	715 245	697 620
	2	13.959	708 727	
	3	13.932	668 887	
0.002445	1	13.934	199 290	192 139
	2	13.938	200 749	
	3	13.959	176 379	
0.000734	1	13.938	71 607	81 348
	2	13.956	85 217	
	3	13.966	87 219	

Supplementary Table S2. Experimental data for the calculation of the linearity curve of dehydroabietic acid

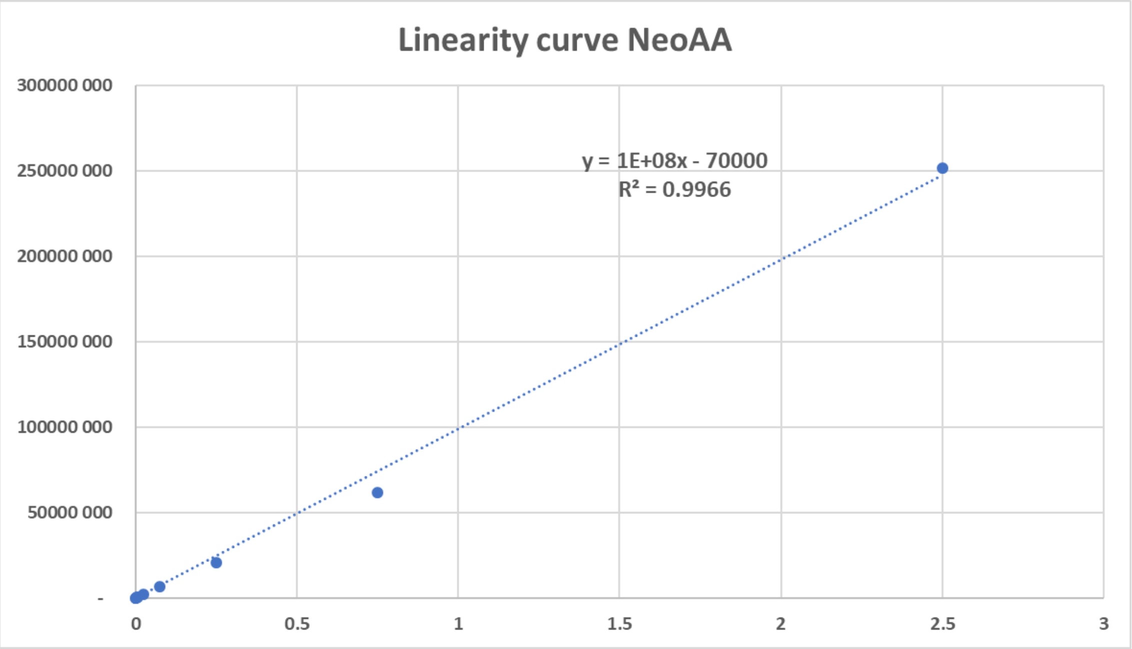

Supplementary Figure S5. Linearity curve of neoabietic acid

c [mg/ml]	Run	RT [min]	Area	Mean
7.5	1	13.917	499795141	498 286 885
	2	13.916	498704548	
	3	13.914	496360967	
2.5	1	13.929	252165844	251 661 129
	2	13.931	251317481	
	3	13.931	251500063	

0.75	1	13.987	62355659	61 879 886
	2	13.990	61778821	
	3	13.996	61505179	
0.25	1	14.042	20672692	20 945 889
	2	14.027	21217309	
	3	14.027	20947667	
0.075	1	14.075	6912575	6 827 319
	2	14.055	6805296	
	3	14.076	6764087	
0.025	1	14.085	2173278	2 145 625
	2	14.087	2162849	
	3	14.091	2100747	
0.0075	1	14.090	731793	729 842
	2	14.109	709099	
	3	14.112	748635	
0.0025	1	14.094	378984	368 469
	2	14.106	368446	
	3	14.092	357978	
0.00075	1	14.107	72901	74 350
	2	14.098	75606	
	3	14.127	74542	

Supplementary Table S3. Experimental data for the calculation of the linearity curve of neoabietic acid

3.3. Specificity

**Reference substances**		**Extr. Ion**	**Extract**		**Deviation**
**Resin acid**	**RT [min]**	**[Da]**	**Peak number**	**RT [min]**	**[min]**
Pimaric acid	12.072	301^-	1	12.160	-0.088
Sandaracopimaric acid*	12.387	301^-	2	12.313	0.074
Palustric acid	12.477	301^-	3	12.508	-0.031
Isopimaric acid	12.792	301^-	4	12.801	-0.009
Levopimaric acid	13.064	301^-	5	12.963	0.101
Abietic acid	13.586	301^-	6	13.575	0.011
Dehydroabietic acid	13.873	299^-	7	13.885	-0.012
Neoabietic acid	14.024	301^-	8	13.975	0.049

*sandarac extract was tested

Supplementary Table S4. References of the resin acids were compared to the respective constituent of *Picea abies* balm extract NSB22. The deviation of the reference substance to the resin acid of the extract was calculated.

3.4. Accuracy

		DHAA Area	Recovery rate
1	NSB22 2mg/ml	20 352 108	
	DHAA 0.183 mg/ml	10 933 543	
	DHAA 0.244 mg/ml	15 036 184	
	DHAA 0.305 mg/ml	18 396 690	
	NSB22 2 mg/ml+DHAA 0.183 mg/ml	32 220 874	**97.1**
	NSB22 2 mg/ml+DHAA 0.244 mg/ml	38 094 500	**92.9**
	NSB22 2 mg/ml+DHAA 0.305 mg/ml	43 683 516	**88.7**
2	NSB22 2mg/ml	19 722 408	
	DHAA 0.183 mg/ml	10 802 031	
	DHAA 0.244 mg/ml	15 237 185	
	DHAA 0.305 mg/ml	17 770 642	
	NSB22 2 mg/ml+DHAA 0.183 mg/ml	31 473 598	**97.0**
	NSB22 2 mg/ml+DHAA 0.244 mg/ml	37 361 626	**93.6**
	NSB22 2 mg/ml+DHAA 0.305 mg/ml	43 739 038	**85.7**
3	NSB22 2mg/ml	19 531 898	
	DHAA 0.183 mg/ml	10 768 794	
	DHAA 0.244 mg/ml	14 706 166	
	DHAA 0.305 mg/ml	17 849 075	
	NSB22 2 mg/ml+DHAA 0.183 mg/ml	31 312 698	**96.8**
	NSB22 2 mg/ml+DHAA 0.244 mg/ml	36 838 298	**92.9**
	NSB22 2 mg/ml+DHAA 0.305 mg/ml	43 734 973	**85.5**
Mean	NSB22 2mg/ml	19 868 805	
	DHAA 0.183 mg/ml	10 834 789	
	DHAA 0.244 mg/ml	14 993 178	
	DHAA 0.305 mg/ml	18 005 469	
	NSB22 2 mg/ml+DHAA 0.183 mg/ml	31 669 057	**97.0**
	NSB22 2 mg/ml+DHAA 0.244 mg/ml	37 431 475	**93.1**
	NSB22 2 mg/ml+DHAA 0.305 mg/ml	43 719 176	**86.6**

Supplementary Table S5. The spruce balm sample NSB22 was spiked with three different concentrations of dehydroabietic acid (DHAA) and analyzed in triplicates. The recovery rate of dehydroabietic acid in the spiked samples was calculated.

		NeoAA Area	Recovery rate
1	NSB22 2 mg/ml	43 792	
	NeoAA_0.0013 mg/ml	66 768	
	NeoAA_0.00174 mg/ml	93 922	
	NeoAA_0.0022 mg/ml	124 029	
	NSB22 2 mg/ml+NeoAA_0.0013 mg/ml	113 330	**102.5**
	NSB22 2 mg/ml+NeoAA_0.00174 mg/ml	161 836	**117.5**
	NSB22 2 mg/ml+NeoAA_0.0022 mg/ml	193 022	**115.0**
2	NSB22 2 mg/ml	41 530	
	NeoAA_0.0013 mg/ml	67 831	
	NeoAA_0.00174 mg/ml	111 900	
	NeoAA_0.0022 mg/ml	148 402	
	NSB22 2 mg/ml+NeoAA_0.0013 mg/ml	121 101	**110.7**
	NSB22 2 mg/ml+NeoAA_0.00174 mg/ml	157 672	**102.8**
	NSB22 2 mg/ml+NeoAA_0.0022 mg/ml	202 629	**106.7**
3	NSB22 2 mg/ml	48 613	
	NeoAA_0.0013 mg/ml	72 781	
	NeoAA_0.00174 mg/ml	102 771	
	NeoAA_0.0022 mg/ml	140 906	
	NSB22 2 mg/ml+NeoAA_0.0013 mg/ml	120 861	**99.6**
	NSB22 2 mg/ml+NeoAA_0.00174 mg/ml	147 631	**97.5**
	NSB22 2 mg/ml+NeoAA_0.0022 mg/ml	194 101	**102.4**
Mean	NSB22 2 mg/ml	44 645	
	NeoAA_0.0013 mg/ml	69 127	
	NeoAA_0.00174 mg/ml	102 864	
	NeoAA_0.0022 mg/ml	137 779	
	NSB22 2 mg/ml+NeoAA_0.0013 mg/ml	118 431	**104.1**
	NSB22 2 mg/ml+NeoAA_0.00174 mg/ml	155 713	**105.6**
	NSB22 2 mg/ml+NeoAA_0.0022 mg/ml	196 584	**107.8**

Supplementary Table S6. The spruce balm sample NSB22 was spiked with three different concentrations of neoabietic acid (NeoAA) and analyzed in triplicates. The recovery rate of neoabietic acid in the spiked samples was calculated.

3.5. Precision – Repeatability

0,625 mg/ml	Run 1		Run 2		Run 3		Mean	SD	RSD
	RT	Area	RT	Area	RT	Area			
1	11.947	100 734	12.000	99 370	12.032	96 899	99 001	1 587	1.6
2	12.122	358 872	12.180	373 379	12.166	384 815	372 355	10 616	2.9
3	12.199	119 338	12.261	105 639	12.256	123 405	116 127	7 600	6.5
4	12.637	552 477	12.676	549 646	12.690	564 260	555 461	6 328	1.1
5	12.719	204 037	12.786	195 530	12.789	198 150	199 239	3 557	1.8
6	13.487	41 983	13.475	38 784	13.522	44 494	41 754	2 337	5.6
7	13.733	4 729 646	13.776	4 777 050	13.768	4 809 858	4 772 185	32 927	0.7
8	13.799	34 033	13.856	30 066	13.836	34 935	33 011	2 115	6.4

1.250 mg/ml	Run 1		Run 2		Run 3		Mean	SD	RSD
	RT	Area	RT	Area	RT	Area			
1	11.743	215 858	11.932	195 089	11.950	211 049	207 332	8 877	4.3
2	11.894	760 452	12.125	804 084	12.132	741 675	768 737	26 143	3.4
3	11.962	277 328	12.199	227 231	12.196	271 967	258 842	22 459	8.7
4	12.420	1 202 828	12.622	1 122 395	12.646	1 191 860	1 172 361	35 614	3.0
5	12.507	378 278	12.726	430 982	12.743	429 799	413 020	24 571	5.9
6	13.213	65 718	13.462	63 592	13.416	71 167	66 826	3 190	4.8
7	13.517	9 894 656	13.749	9 827 699	13.751	10 048 112	9 923 489	92 264	0.9
8	13.603	65 470	13.810	62 235	13.820	75 362	67 689	5 584	8.2

1.875 mg/ml	Run 1		Run 2		Run 3		Mean	SD	RSD
	RT	Area	RT	Area	RT	Area			
1	11.926	313 752	11.927	339 717	11.921	316 352	323 274	11 676	3.6
2	12.099	1 120 618	12.100	1 112 769	12.108	1 128 706	1 120 698	6 506	0.6
3	12.156	473 630	12.159	429 758	12.169	397 591	433 660	31 165	7.2
4	12.652	1 933 482	12.616	1 777 968	12.626	1 824 312	1 845 254	65 192	3.5
5	12.719	517 400	12.706	574 492	12.716	554 283	548 725	23 637	4.3
6	13.391	108 108	13.420	101 959	13.417	124 673	111 580	9 592	8.6
7	13.722	15 403 853	13.708	15 179 091	13.716	14 747 474	15 110 139	272 365	1.8
8	13.793	99 771	13.783	102 051	13.779	108 963	103 595	3 908	3.8

Supplementary Table S7. Three different concentrations of sample NSB22 were analyzed in triplicates to assess the repeatability. The relative standard deviation was calculated for each resin acid at each concentration (pimaric acid (**1**), sandaracopimaric acid (**2**), palustric acid (**3**), isopimaric acid (**4**), levopimaric acid (**5**), abietic acid (**6**), dehydroabietic acid (**7**), and neoabietic acid (**8**))

3.5. Precision - Intermediate Precision

1,875 mg/ml	1 - Day 1		1 - Day 2		1 - Day 3		Mean	SD	RSD
	RT	Area	RT	Area	RT	Area			
1	12.233	351 699	12.221	339 092	12.169	307 323	332 705	18 671	5.6
2	12.399	1 550 033	12.390	1 462 874	12.329	1 197 898	1 403 602	149 743	10.7
3	12.530	255 956	12.586	252 296	12.479	221 569	243 274	15 420	6.3
4	12.876	3 151 372	12.881	2 994 062	12.803	2 469 172	2 871 535	291 672	10.2
5	13.080	147 524	13.076	269 641	12.956	255 158	224 108	54 475	24.3
6	13.648	206 573	13.669	215 831	13.573	151 821	191 408	28 246	14.8
7	13.961	16 786 248	13.956	15 400 355	13.881	13 284 812	15 157 138	1 439 764	9.5
8	14.026	96 778	14.026	91 851	13.947	71 104	86 578	11 125	12.8

1,875 mg/ml	2 - Day 1		2 - Day 2		2 - Day 3		Mean	SD	RSD
	RT	Area	RT	Area	RT	Area			
1	12.275	360 760	12.213	354 763	12.161	262 696	326 073	44 881	13.8
2	12.439	1 477 809	12.378	1 407 038	12.337	1 184 938	1 356 595	124 771	9.2
3	12.574	254 729	12.516	258 742	12.463	250 286	254 586	3 454	1.4
4	12.936	3 186 087	12.883	2 923 543	12.845	2 457 941	2 855 857	301 093	10.5
5	13.120	170 620	13.056	247 813	13.026	225 742	214 725	32 462	15.1
6	13.738	203 439	13.647	174 961	13.598	169 427	182 609	14 901	8.2
7	14.018	16 141 384	13.963	15 426 084	13.929	12 893 322	14 820 263	1 393 495	9.4
8	14.093	94 815	14.020	94 711	13.979	78 992	89 506	7 435	8.3

1,875 mg/ml	3 - Day 1		3 - Day 2		3 - Day 3		Mean	SD	RSD
	RT	Area	RT	Area	RT	Area			
1	12.261	338 056	12.211	308 585	12.183	289 265	311 969	20 062	6.4
2	12.429	1 265 966	12.370	1 397 354	12.367	1 107 744	1 257 021	118 402	9.4
3	12.569	247 782	12.501	269 735	12.487	216 667	244 728	21 772	8.9
4	12.943	2 735 245	12.873	2 895 296	12.863	2 277 077	2 635 873	261 986	9.9
5	13.143	143 091	13.042	243 962	13.027	245 517	210 857	47 922	22.7
6	13.715	190 935	13.651	206 293	13.630	169 931	189 053	14 904	7.9
7	14.092	14 212 017	13.954	15 477 498	13.936	11 927 762	13 872 426	1 468 933	10.6
8	14.092	75 108	14.023	89 201	13.990	75 734	80 014	6 501	8.1

Supplementary Table S8. Spruce balm sample NSB22 was analyzed at a concentration of 1,875 mg/ml in triplicates over three days to assess the intermediate precision (pimaric acid (**1**), sandaracopimaric acid (**2**), palustric acid (**3**), isopimaric acid (**4**), levopimaric acid (**5**), abietic acid (**6**), dehydroabietic acid (**7**), and neoabietic acid (**8**)). Table S10 summarizes the mean values, SD and RSD of all intermediate precision – experiments

All experiments	Mean	SD	RSD
**1**	323 582	31 568	9.8
**2**	1 339 073	145 159	10.8
**3**	247 529	16 325	6.6
**4**	2 787 755	305 009	10.9
**5**	216 563	46 226	21.3
**6**	187 690	20 685	11.0
**7**	14 616 609	1 534 054	10.5
**8**	85 366	9 461	11.1

Supplementary Table S9. The results of the intermediate precision experiments are summarized. The mean value, SD and RSD are calculated for each resin acid (pimaric acid (**1**), sandaracopimaric acid (**2**), palustric acid (**3**), isopimaric acid (**4**), levopimaric acid (**5**), abietic acid (**6**), dehydroabietic acid (**7**), and neoabietic acid (**8**))**.**

3.6. Detection limit (LOD) and Quantitation limit (LOQ)

#	RT [min]	Height	#	RT [min]	Height	#	RT [min]	Height
1	1.007	1965	6	6.045	1149	11	11.050	1404
2	2.010	1196	7	7.003	1479	12	12.005	1061
3	2.996	2100	8	7.971	1063	13	13.072	2042
4	4.006	1479	9	9.011	1576	14	15.126	1296
5	5.042	1106	10	10.067	1238	15	15.927	1641
							**Mean:**	**1453**

Supplementary Table S10. Noise value at 10 different time points of a solvent blank for the calculation of the signal/noise ratio of dehydroabietic acid

	c [mg/ml]	height	S/N factor	Mean
**LOD**	**0.00075**	8661	6.0	**5.7**
		8376	5.8	
		7807	5.4	
**LOQ**	**0.0025**	18403	12.7	**13.4**
		19554	13.5	
		20432	14.1	

Supplementary Table S11. Limit of detection (LOD) and limit of quantitation (LOQ) of dehydroabietic acid

#	RT [min]	Height	#	RT [min]	Height	#	RT [min]	Height
1	1.036	1073	6	6.011	1726	11	10.979	1124
2	1.976	1109	7	7.010	1577	12	11.979	1276
3	2.893	1127	8	7.943	865	13	13.000	1572
4	3.979	1317	9	9.009	1440	14	15.004	1085
5	5.068	1707	10	10.031	1154	15	15.996	1280
							**Mean:**	**1295**

Supplementary Table S12. Noise value at 10 different time points of a solvent blank for the calculation of the signal/noise ratio of neoabietic acid

	c [mg/ml]	height	S/N factor	Mean
**LOD**	0.001	6269	4.8	**5.0**
		5553	4.3	
		7690	5.9	
**LOQ**	0.002	11294	8.7	**9.3**
		12484	9.6	
		12475	9.6	

Supplementary Table S13. Limit of detection (LOD) and limit of quantitation (LOQ) of neoabietic acid

4. Quantitation

	**1**	**2**	**3**	**4**	**5**	**6**	**7**	**8**	Total
Ret.-time ([min])	12.150±0.03	12.316±0.02	12.518±0.03	12.812±0.05	13.065±0.05	13.629±0.04	13.931±0.04	14.058±0.05	
NSB01	0.3 (±0.005)	1.3 (±0.038)	3.8 (±0.026)	3.4 (±0.046)	4.6 (±0.089)	2.4 (±0.072)	7.1 (±0.051)	2.3 (±0.049)	25.1 (±0.181)
NSB02	0.3 (±0.006)	1.4 (±0.011)	3.1 (±0.042)	2.5 (±0.023)	4.9 (±0.02)	2.0 (±0.015)	7.7 (±0.054)	1.8 (±0.045)	23.7 (±0.122)
NSB03	0.3 (±0.011)	1.5 (±0.012)	0.2 (±0.016)	2.2 (±0.004)	0.02 (±0.021)	0.5 (±0.008)	10.5 (±0.062)	0.0 (±0.004)	15.3 (±0.076)
NSB04	0.3 (±0.012)	1.1 (±0.010)	2.7 (±0.019)	3.2 (±0.031)	3.5 (±0.003)	1.8 (±0.024)	10.2 (±0.133)	1.0 (±0.034)	23.8 (±0.187)
NSB05	0.4 (±0.018)	1.6 (±0.038)	4.5 (±0.118)	3.7 (±0.043)	7.8 (±0.060)	2.6 (±0.032)	5.7 (±0.046)	3.1 (±0.037)	29.3 (±0.270)
NSB06	0.4 (±0.006)	1.6 (±0.03)	4.7 (±0.007)	3.7 (±0.044)	10.8 (±0.108)	2.0 (±0.158)	3.8 (±0.023)	3.9 (±0.135)	31.0 (±0.415)
NSB07	0.2 (±0.007)	1.5 (±0.028)	3.2 (±0.056)	2.7 (±0.042)	6.9 (±0.021)	2.8 (±0.004)	5.3 (±0.062)	3.6 (±0.025)	26.3 (±0.062)
NSB08	0.3 (±0.020)	1.9 (±0.031)	3.2 (±0.041)	3.3 (±0.020)	6.9 (±0.032)	2.0 (±0.020)	9.1 (±0.044)	2.5 (±0.019)	29.1 (±0.077)
NSB09	0.4 (±0.008)	1.3 (±0.031)	0.5 (±0.030)	2.0 (±0.020)	0.4 (±0.018)	0.4 (±0.009)	10.3 (±0.085)	0.1 (±0.008)	15.4 (±0.125)
NSB10	0.6 (±0.016)	2.0 (±0.032)	4.1 (±0.032)	3.5 (±0.013)	10.4 (±0.047)	2.4 (±0.040)	5.6 (±0.002)	3.7 (±0.053)	32.2 (±0.059)
NSB11	0.5 (±0.003)	2.0 (±0.047)	4.0 (±0.006)	2.5 (±0.053)	9.2 (±0.076)	1.6 (±0.194)	5.2 (±0.019)	2.4 (±0.188)	27.4 (±0.556)
NSB12	0.4 (±0.011)	1.5 (±0.022)	5.5 (±0.013)	2.0 (±0.049)	30.8 (±0.138)	1.9 (±0.059)	1.7 (±0.017)	3.1 (±0.085)	46.9 (±0.275)
NSB13	0.1 (±0.034)	0.6 (±0.132)	0.2 (±0.077)	1.4 (±0.315)	0.1 (±0.017)	0.3 (±0.093)	5.6 (±1.217)	0.0 (±0.002)	8.4 (±1.879)
NSB14	0.2 (±0.004)	0.8 (±0.014)	1.1 (±0.004)	3.8 (±0.027)	0.5 (±0.043)	1.4 (±0.007)	6.8 (±0.052)	0.4 (±0.006)	15.0 (±0.094)
NSB15	0.2 (±0.016)	1.3 (±0.028)	0.3 (±0.018)	1.7 (±0.027)	0.1 (±0.022)	0.2 (±0.009)	9.0 (±0.075)	0.0 (±0.004)	12.8 (±0.078)
NSB16	0.2 (±0.022)	1.2 (±0.018)	2.2 (±0.025)	2.1 (±0.045)	5.8 (±0.068)	1.6 (±0.133)	5.3 (±0.034)	1.6 (±0.118)	20.0 (±0.360)
NSB17	0.4 (±0.016)	1.3 (±0.024)	2.2 (±0.034)	2.3 (±0.030)	3.9 (±0.121)	1.1 (±0.067)	6.7 (±0.034)	1.2 (±0.025)	19.1 (±0.324)
NSB18	0.4 (±0.013)	1.6 (±0.016)	2.8 (±0.035)	2.8 (±0.017)	0.6 (±0.004)	3.3 (±0.011)	8.0 (±0.019)	0.8 (±0.4)	20.2 (±0.348)
NSB19	0.7 (±0.008)	1.6 (±0.013)	0.2 (±0.016)	1.7 (±0.062)	0.1 (±0.021)	0.2 (±0.006)	9.3 (±0.032)	0.0 (±0.002)	13.8 (±0.133)
NSB20	0.6 (±0.01)	1.9 (±0.041)	1.9 (±0.016)	3.3 (±0.046)	2.0 (±0.071)	1.4 (±0.038)	8.8 (±0.032)	0.9 (±0.016)	20.9 (±0.203)
NSB21	0.5 (±0.005)	2.3 (±0.025)	2.6 (±0.064)	3.6 (±0.025)	3.2 (±0.001)	2.3 (±0.022)	7.8 (±0.012)	1.8 (±0.017)	24.0 (±0.070)
NSB22	0.2 (±0.009)	0.8 (±0.01)	0.3 (±0.01)	1.9 (±0.01)	0.1 (±0.033)	0.2 (±0.007)	9.2 (±0.112)	0.0 (±0.001)	12.7 (±0.132)
Mean	0.4 (±0.150)	1.5 (±0.398)	2.5 (±1.612)	2.7 (±0.743)	5.2 (±6.601)	1.6 (±0.908)	7.2 (±2.215)	1.6 (±1.316)	22.7 (±8.404)

	**1**	**2**	**3**	**4**	**5**	**6**	**7**	**8**	Total
Ret.-time ([min])	12.150±0.03	12.316±0.02	12.518±0.03	12.812±0.05	13.065±0.05	13.629±0.04	13.931±0.04	14.058±0.05	
NSB-CP1	n.d.	n.d.	0.02 (±0.001)	0.07 (±0.001)	0.11 (±0.002)	0.15 (±0.005)	0.28 (±0.008)	0.05 (±0.007)	0.64 (±0.014)
NSB-CP 2	n.d.	n.d.	0.02 (±0.001)	0.08 (±0.002)	0.11 (±0.004)	0.17 (±0.003)	0.26 (±0.005)	0.06 (±0.002)	0.70 (±0.006)
NSB-CP 3	n.d.	n.d.	0.03 (±0.001)	0.08 (±0.001)	0.12 (±0.004)	0.17 (±0.004)	0.26 (±0.004)	0.07 (±0.002)	0.73 (±0.009)
NSB-CP4	0.05 (±0.004)	0.24 (±0.009)	0.79 (±0.016)	0.48 (±0.007)	1.9 (±0.012)	0.53 (±0.009)	0.57 (±0.005)	0.71 (±0.021)	5.27 (±0.052)
NSB-CP5	0.06 (±0.003)	0.26 (±0.011)	0.81 (±0.008)	0.50 (±0.014)	2.46 (±0.026)	0.48 (±0.013)	0.5 (±0.005)	0.82 (±0.026)	5.88 (±0.049)

Supplementary Table S14. Retention times (min ±SD), contents and total contents (% ±SD) of the eight diterpene acids (pimaric acid (**1**), sandaracopimaric acid (**2**), palustric acid (**3**), isopimaric acid (**4**), levopimaric acid (**5**), abietic acid (**6**), dehydroabietic acid (**7**), and neoabietic acid (**8**)) in Norway Spruce Balm (samples NSB01-22) and commercial products (NSB-CP1-5). Contents of compounds **1-6** were calculated as neoabietic acid (**8**).
